# Severe renal dysfunction measured by pRIFLE in children is an independent factor associated with death and not predicted by PIM2

**DOI:** 10.1186/cc12347

**Published:** 2013-03-19

**Authors:** J Piva, P Lago, G Pedro, F Cabral

**Affiliations:** 1 UFRGS, Porto Alegre, Brazil; 2 Hospital de Clinicas de Porto Alegre, Brazil; 3 PUCRS, Porto Alegre, Brazil

## Introduction

The objective was to evaluate the effect of the renal dysfunction measured by pRIFLE on clinical outcome in children admitted to the pediatric intensive care unit (PICU).

## Methods

A retrospective cohort involving all children admitted between August 2009 and July 2010 to a Brazilian PICU located in a university-affiliated hospital. Patients were classified according to the pRIFLE at admission as well the maximum pRIFLE during the PICU stay. The endpoints were: length of stay (LOS) in the PICU, LOS on mechanical ventilation, mortality rate quoted by predicted mortality of the Pediatric Index of Mortality (PIM2).

## Results

In total, 375 children were included in the study. A total 169 (45%) of them presented renal dysfunction according to the pRIFLE. Renal injury and renal failure were associated with higher LOS in the PICU and length of mechanical ventilation. The observed and predicted mortality by PIM2 did not show differences. However, the more severe renal presentation (Injury and Failure) was associated with higher mortality rate and was not predicted by PIM2 (8% vs. 11%, *P *0.01).

## Conclusion

Renal dysfunction measured by pRIFLE is a very common finding (45%) among pediatric patients admitted to the PICU. The more severe renal affection is associated with higher mortality, length of MV and length of PICU stay.
